# Racial Regeneration

**Published:** 1898-09

**Authors:** F. B. Ambler


### Racial Regeneration.

The unexpected has happened. The races cradled in the warm bosom of the future are to be elevated by the practical wisdom of the present age. From the abstracted cerebrations of the man of science, to a practical recognition of their utility, as applicable to the problem of racial regeneration, is a long step and yet it has remained for a layman to inaugurate the definite features of a plan, operating along feasible and lawful lines to accomplish this exalted end. The reconstructive forces of society are to be marshalled in offensive array, the maudlin passions of humanity are to be subdued, the mandates of statuatory law are to be invoked to assort properly all candidates for matrimony; the infirm and tainted are to be denied this boon under penalty of the law and henceforth babies, like calves, are to be bred by human selection.

Though the work of a layman, this plan at once so logical and novel, embodies considerations of exact scientific importance and rests its affirmation upon the broad principles of natural law. In brief its supporters contemplate the establishment of legislative enactments in all the States of the nation providing that candidates for matrimony, shall in accordance with the possession or absence of certain physical conditions, indispensable to a sound unvitiated organism, be accepted or rejected as the case may be. This measure has already been introduced into one of the legislative assemblies of the nation and the coming year promises to see it a burning question wherever "Solons" congregate. Necessarily influence of medical men everywhere will be solicited and their opinions valued; hence the premature expression of an opinion on my part will not be out of place. It can be well said that it is an attempt at legislation at once insane and yet sane. It will not work perhaps, and yet it ought to. The civilizing tendencies of a race rest upon a handful of fundamental ideas. These ideas are slowly evolved and as tardily disappear. Though they be manifestly foolish and absurd to the erudite, to the masses they are manifest truths. The masses live in the past, cherish theories that have been dissipated, confide in faiths that are gone. In consequence there is an obvious difficulty in eradicating old ideas as there is equal difficulty in establishing the new. Both require often long periods and stormy cataclysms, generally revolutions. In the vocations of life which are the arena of our activities (and in which we shirk all the duties we can) it has been an unwritten law and a fundamental idea that every influence should be exerted to foster and encourage matrimony, regardless of any factors of fitness, ignoring any other considerations of quality, driven by blind passion or a capricious fate. Anything deterrent to matrimony is considered an abridgement to personal liberty and adverse to public policy. This idea is more than a sentiment,—it is a law. The practical workings of this unwritten law has been the creation of hospitals and prisons, and the world teems with spawn of vitiated organisms and ill-assorted unions. Should a bill of this character be enacted and its provisions be established throughout the land these places of detention and rehabilitation would soon constitute the monuments of an unregretted past. We would witness the end of disease and the end of crime. Of course such a future is a chimera and a dream judged in the light of present conditions, hence an enactment of such a character would be incompetent and insufficient to meet its logical expectations. It cannot be considered in the light of a question of statute but of sentiment entirely. The idea must first become fundamental, and from a theory we must develop an instinct that shall be strong enough to dominate the race.

The evolution of humanity has proceeded along such lines, its onward march has been punctuated by such fundamental processes, and practices recognized as ethical, alter as intelligence spreads. There was a time when miscegenation was legitimate, and other crimes of the same category were matters of no moment. Today they are abhorent and infrequent. It is not enactments that have made them so. Their infrequence is due to an idea, a conviction, feeble and blind at first, yet growing bolder and more obstinate with time, and gaining strength that when transmitted becomes an inherited repulsion. This reform movement may not be even a step in this much desired evolution, and yet it demands our respect as the germinal principle of a potentiality which will permeate the crust of sociological conditions, find its proper soil, take root, grow and blossom into one of those exalted instincts, shared by all civilized men. Should we ever attain this ideal state, we may confidently look toward a race freed from the fetters of disease, devoid of the instincts of crime, and animated by fresh zeal, new instincts and senses, and an enthusiasm that shall win victories that even the most ardent votaries of science do not dream.—F. B. Ambler, in *Colorado Medical Journal.*

